# Digital Teleconsultation: Clinical Perspectives

**DOI:** 10.1155/2014/460989

**Published:** 2014-12-23

**Authors:** David Wilbur

**Affiliations:** Massachusetts General Hospital, Boston, MA 03076, USA

## Introduction

The emerging field of digital pathology has many applications including the ability to easily and quickly consult with colleagues at a distance.

## Methods

Teleconsultation can take a variety of forms and has a number of different applications.

## Results

Immediate analysis of specimens, such as rapid cytology adequacy determination and frozen section interpretation are now employed using real time screen sharing and whole slide imaging technologies. Both have been shown to be accurate and have proven to be substantial productivity enhancers for pathologists. Secondary consultation practices have employed predominantly the whole slide imaging approach and have also been shown to be accurate. Uptake of teleconsultation for second opinions has been limited by the expense and therefore availability of scanning technology, but in the future, as whole slide imaging become more routine and cost-effective, this aspect of teleconsultation is certain to grow.

## Conclusions

This presentation will discuss the workflow and components necessary for the types of teleconsultation noted above. Available data in support of each application will be reviewed. Practical considerations for implementation and pitfalls will be discussed.

